# Controlled on-chip fabrication of large-scale perovskite single crystal arrays for high-performance laser and photodetector integration

**DOI:** 10.1038/s41377-023-01107-4

**Published:** 2023-03-08

**Authors:** Zhangsheng Xu, Xun Han, Wenqiang Wu, Fangtao Li, Ru Wang, Hui Lu, Qiuchun Lu, Binghui Ge, Ningyan Cheng, Xiaoyi Li, Guangjie Yao, Hao Hong, Kaihui Liu, Caofeng Pan

**Affiliations:** 1 CAS Center for Excellence in Nanoscience, Beijing Key Laboratory of Micro-nano Energy and Sensor, Beijing Institute of Nanoenergy and Nanosystems, Chinese Academy of Sciences, Beijing, 101400 China; 2 School of Nanoscience and Technology, University of Chinese Academy of Sciences, Beijing, 100049 China; 3 College of Mechatronics and Control Engineering, Shenzhen University, Shenzhen, 518060 China; 4 College of Physics and Optoelectronic Engineering, Shenzhen University, Shenzhen, 518060 China; 5 Center on Nanoenergy Research, School of Physical Science and Technology, Guangxi University, Nanning, 530004 China; 6 Information Materials and Intelligent Sensing Laboratory of Anhui Province, Key Laboratory of Structure and Functional Regulation of Hybrid Materials of Ministry of Education, Institutes of Physical Science and Information Technology, Anhui University, Hefei, 230601 China; 7 College of Materials Science and Engineering, Ocean University of China, Qingdao, 266100 China; 8 State Key Laboratory for Mesoscopic Physics, Frontiers Science Centre for Nano-optoelectronics, School of Physics, Peking University, Beijing, 100871 China; 9 Songshan Lake Materials Laboratory, Dongguan, 523808 China

**Keywords:** Optical materials and structures, Photonic devices

## Abstract

Metal halide perovskites possess intriguing optoelectronic properties, however, the lack of precise control of on-chip fabrication of the large-scale perovskite single crystal arrays restricts its application in integrated devices. Here, we report a space confinement and antisolvent-assisted crystallization method for the homogeneous perovskite single crystal arrays spanning 100 square centimeter areas. This method enables precise control over the crystal arrays, including different array shapes and resolutions with less than 10%-pixel position variation, tunable pixel dimensions from 2 to 8 μm as well as the in-plane rotation of each pixel. The crystal pixel could serve as a high-quality whispering gallery mode (WGM) microcavity with a quality factor of 2915 and a threshold of 4.14 μJ cm^−2^. Through directly on-chip fabrication on the patterned electrodes, a vertical structured photodetector array is demonstrated with stable photoswitching behavior and the capability to image the input patterns, indicating the potential application in the integrated systems of this method.

## Introduction

Metal halide perovskites have demonstrated promising applications in photovoltaics due to their superior optoelectronic properties^1–3^ and solution-based fabrication processes^4–10^. For example, the certified efficiency of the perovskite solar cell has achieved a remarkable evolution from 14.1 to 25.8%^11–13^ over the past decade. Inspired by the great success in photovoltaics, perovskite-based optoelectronic devices, including photodetectors^14–19^, light-emitting diodes^20–24^, lasers^7,8,25–27^, and field-effect transistors^28–30^, have been developed with significantly enhanced performance, which could bring the paradigm shift of the device design in the traditional optoelectronic industry. Most of these devices adopt the continuous polycrystalline films as the active layer that are typically widely demanded in the photovoltaics community through the large-area coating method^31–35^. However, different from photovoltaic devices, a large number of optoelectronic devices require discrete perovskite layers for functional integrated applications. To further improve the performance of the perovskite devices, it is crucial to develop perovskite single-crystal arrays with excellent chemical stability, lower defect density, and higher carrier mobility^36–39^.

Due to the poor chemical stability of the perovskite materials in the polar solvent, the most commonly used photolithography and etching techniques for patterning the single crystal arrays are highly incompatible with these materials. In this regard, various efforts to fabricate the perovskite single-crystal arrays have been demonstrated with the two-step vapor-phase method containing the deposition of precursor seed array and converting step in the furnace^14,40,41^. Other strategies have focused on the one-step fabrication process to separate the precursor domains through the template with microstructures^7^ and capillary force^5,17,42^ or employing the inkjet printing^43^ to directly print the precursor array on the desired substrate followed by the controlled crystallization at a certain temperature. However, these methods are suitable for crystal synthesis on a blank substrate. No method has yet to be demonstrated for the on-chip fabrication to realize alignment between the perovskite array and as-fabricated patterns with precise control of individual pixel properties. Such perovskite arrays and capabilities, not only enable the fabrication of the devices based on the individual crystals but also demonstrate direct integration with large-scale optoelectronic devices.

In this work, we develop a one-step space confinement and antisolvent-assisted crystallization (SC-ASC) method which can fabricate high-quality single-crystalline MAPbBr_*x*_Cl_3-*x*_ (MA = CH_3_NH_3_, *x* = 0, 1, 2, 3) microplate (MP) arrays on the various substrates. This method provides comprehensive control over the MPs array, including the array shape and resolution with superior crystal dimension and position accuracy as well as the in-plane rotation of the individual MPs in a large-scale array configuration. The keys to realizing fully controlled growth are (1) substrate engineering to ensure accurate pixel positioning; (2) space confinement to regulate the extraction of contact line without leaving residue; and (3) antisolvent crystallization to improve the crystal quality. The perovskite MP can be served as a high-quality microcavity for WGM laser emission with a high Q factor of 2915 and low threshold of 4.14 μJ cm^−2^, and the lasing mode can be tuned by the dimensions of MPs. Due to the readily regulation of the perovskite MPs array as well as its excellent uniformity, a perovskite photodetector array based on the vertical structure has been demonstrated, showing a stable dynamic photobehavior. The input patterns can be easily obtained by mapping the output current from all pixels. Therefore, this approach for the fully controlled growth of perovskite array combining the conventional photolithography and solution processing procedures of perovskites offers a manufacturing platform for integrated functional photonics and optoelectronics.

## Results

### Fully-controlled fabrication of single-crystalline perovskite MPs array

**Fig. 1:**
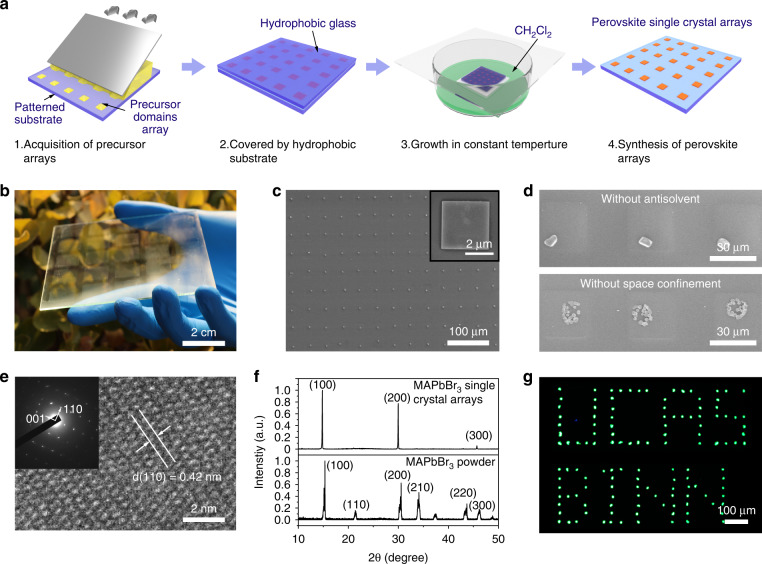
Fabrication process and structural characterization of perovskite MPs array. **a** Schematic illustration for the synthesis process of perovskite MPs array. **b** Optical image of the MAPbBr_3_ MPs array fabricated on the 10 × 10 cm glass. **c** SEM image of MAPbBr_3_ MPs array. The inset is an enlarged view of an individual MAPbBr_3_ MP. **d** SEM images of MAPbBr_3_ single crystal array fabricated without antisolvent (top) and space confinement (bottom). **e** HRTEM image of a single MAPbBr_3_ MP. The inset is SAED patterns. **f** X-ray diffraction of as-synthesized MAPbBr_3_ MPs array and MAPbBr_3_ powder. **g** Fluorescence micrograph of the MAPbBr_3_ MPs array under 365 nm light illumination showing the bright green pattern of the “BINN” and “UCAS”

Figure 1a illustrates the fabrication steps of the single-crystalline MPs array. We previously reported a fabrication process for the pre-patterned SiO_2_ substrate through surface functionalization^10^, which can generate a hydrophilic area array on a hydrophobic substrate surface. Here, we modified this method and adopted it to various substrates for hydrophilic patterns, including metal electrodes, indium tin oxide (ITO) film, and polyethylene glycol terephthalate (PET) substrate (Fig. S1). The precursor solution was restricted in the hydrophilic areas of the pre-patterned substrate (target substrate) in the drop-casting process and then it was confined by a hydrophobic glass (confinement substrate) in the vertical direction (Fig. 1a). The gap between the two substrates is fixed at around 20 μm (Fig. S2). Subsequently, an antisolvent-assisted crystallization strategy was adopted to improve the crystalline quality. The growth of perovskite crystals was monitored, showing that with the extraction of the contact line, the square-shaped perovskite MPs were formed in the center of the regular domain arrays (Fig. S3). Owing to the hydrophobic surface of the top confinement glass, these MPs were located on the target substrate with a well-aligned customized pattern (detailed see Method).

SC-ASC is an effective method for the fabrication of large-scale single-crystalline perovskite MPs array due to that it combines the conventional photolithography process and antisolvent crystallization with space confinement. To demonstrate the large-scale perovskite MPs array, a 10 cm-wide pre-patterned substrate with a hydrophilic area width of 20 μm was utilized. Figure 1b depicts the single-crystalline MAPbBr_3_ MPs array on a 10 × 10 cm glass substrate, consisting of 25 perovskite array patterns and 1250 × 1250 pixels in total. The detailed morphology of the perovskite array was characterized by the scanning electron microscope (SEM), showing that the MPs are well arranged on the substrate in a homogeneous square shape with a width of 5 μm (Fig. 1c). The pitch of the MPs array is well consistent with the designed geometries of hydrophilic and hydrophobic areas. Smooth surfaces and sharp edges are also observed in the magnified and tilted SEM images (Fig. S4), confirming the high crystalline quality of the perovskite MPs array. It is worth noting that the antisolvent and space confinement demonstrated a significant influence on the crystal growth behavior of the perovskite MPs array. As shown in Fig. 1d, the MPs array with irregular shapes was obtained under the antisolvent-free condition, indicating the uncontrollable growth of perovskite crystals. The absence of space confinement could speed up solvent evaporation, leading to the pining of the contact line, and thereby yield the randomly distributed small grains. The structure of the perovskite MPs array was investigated by transmission electron microscopy (TEM) and X-ray diffraction (XRD). Figure 1e shows the high-resolution TEM (HRTEM) image, presenting a set of (110) planes with a distance of 0.42 nm. The selected area electron diffraction (SAED) pattern illustrates a single set of sharp diffraction spots, which can be indexed to the cubic MAPbBr_3_ structures. The XRD results are summarized in Fig. 1f. The perovskite MPs array presents two strong diffraction peaks at 14.91° and 30.16°, which are ascribed to (100) and (200) planes, respectively. These two peaks are consistent with the reported works^22^ about cubic MAPbBr_3_ perovskite. The TEM and XRD results confirm the single-crystalline structure of the perovskite MPs array. The fluorescence image and the photoluminescence (PL) spectroscopy of the MPs array were obtained under a UV light (365 nm) excitation. A uniform fluorescence from each pixel of four MAPbBr_3_ MPs array was captured, confirming the reliability of this approach for the fabrication of large-scale perovskite arrays (Fig. S5). By exquisitely designing the distribution of the hydrophilic areas on the pre-patterned substrate, various perovskite MPs array patterns could be demonstrated. For example, the “UCAS” and “BINN” shaped patterns have been successfully generated, showing the potential application in integrated optoelectronic devices (Fig. 1g).

**Fig. 2:**
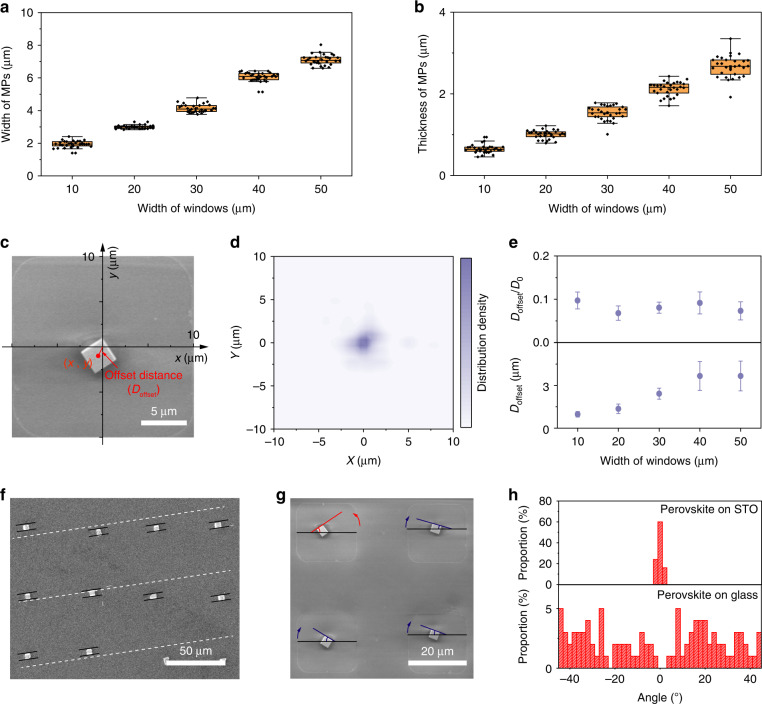
Demonstration of the controlled growth of perovskite MPs array. The dependence of **a** width and **b** thickness of perovskite MPs on the width of the hydrophilic window. **c** Coordinate system of a perovskite MP in a hydrophilic window with a width of 20 μm. **d** Position distribution of 100 perovskite MPs in the coordinate system. **e** The dependence of D_offset_ and D_offset_/D_0_ on the width of the hydrophilic window. **f** SEM image of CsPbBr_3_ MPs array synthesized on STO (100) substrate. **g** Schematic of two rotation angles of perovskite MPs considering the clockwise rotation as positive direction (blue) and the counterclockwise rotation as negative (red). **h** Statistics of the in-plane rotation of 100 perovskite MPs array synthesized on glass and STO (100) substrate

In addition to the controllable pixel patterns, the SC-ASC strategy is versatile and offers plenty of freedom for the fabrication of perovskite array, referring to the precise control of pixel dimension and position, applicability to various substrates, and tunability of in-plane rotation of the MPs. At a certain precursor concentration, the volume of MP is determined by the amount of the precursor domains. Thus, the width and thickness of the MPs can be tuned by changing the width of the window of the hydrophilic area. Perovskite single-crystal arrays with the MP width ranging from 2 to 8 μm and thicknesses ranging from 500 nm to 2.5 μm were demonstrated, showing a linear relationship with the width of the hydrophilic area (Fig. 2a, b). Moreover, both the width and thickness of the MPs were distributed in a narrow range. For example, the thickness and width of 100 perovskite MPs synthesized in the hydrophilic area with a window width of 20 μm were distributed in a narrow range of 2.4 to 3 μm and 0.9 to 1.4 μm, respectively (Fig. S6). To confirm the homogeneous position distribution of the perovskite MPs in the hydrophilic area, we established a rectangular coordinate system with the origin point placed in the center of the hydrophilic window. Using the perovskite MPs array with the hydrophilic window width of 20 μm as an example, the offset distance (D_offset_) was defined as the distance between the center of perovskite MP and the origin point (Fig. 2c). The distribution density of 100 perovskite MPs in the hydrophilic window was calculated by mapping the position of each pixel (Fig. 2d). It demonstrates that these pixels are concentratedly located around the center of the corresponding hydrophilic areas. We also calculated the average of the D_offset_ of 100 perovskite MPs in the hydrophilic areas with different window widths (D_0_) of 20, 30, 40, and 50 μm. As shown in Fig. 2e, with the broadening of the hydrophilic window, the D_offset_ increased slightly from 1 μm to 4 μm. However, the deviation ratio of different hydrophilic areas, defined as D_offset_/D_0_, was maintained below 10%, indicating the capability of precise positioning of each perovskite pixel of this SC-ASC method.

Benefiting from the low-temperature procedures of the SC-ASC method, single-crystalline perovskite MPs have been fabricated on various substrates, including the rigid glass substrate covered by Cr, ITO or NiO film, and the flexible PET substrate (Fig. S7). These perovskite MPs were demonstrated in a square shape with a well-aligned pattern and emitted bright green fluorescence under UV light excitation. We further expanded the SC-ASC method to the oxide perovskite SrTiO_3_ (STO) substrate to regulate the in-plane rotation of the perovskite MPs array. The STO substrate has been reported to be utilized for the epitaxial growth of the cubic CsPbBr_3_ film^44^. There is a big lattice constant mismatch between the STO (100) substrate with the cubic CsPbBr_3_, however, the lattice constant of the CsPbBr_3_ is approximate to 1.5 times that of STO, offering the possibility of epitaxial growth of the CsPbBr_3_ crystal on the STO substrate (Fig. S8). Figure 2f illustrates the SEM image of the CsPbBr_3_ crystal array epitaxially synthesized on the STO substrate. A square shape and sharp edges are also observed in the CsPbBr_3_ MPs array. In addition to the precisely controlled pixel position, the edges of different pixels were well aligned, indicating the epitaxial growth of the CsPbBr_3_ MPs. To reveal the rotation angle distribution of the CsPbBr_3_ MPs array, the in-plane rotation of 100 perovskite MPs on the glass and STO substrate was counted in the manner that the rotation in clockwise (blue line) was considered as the positive direction, while the counterclockwise (red line) rotation was negative (Fig. 2g). Figure 2h presents the comparison of the rotation angle of perovskite MPs array fabricated on the STO and glass substrate. The rotation angles of perovskite MPs fabricated on the STO were restricted to a narrow range from −2° to 2° and 60% of MPs did not demonstrate rotation, whereas perovskite MPs fabricated on the glass were randomly distributed over the entire range. Besides, KTaO_3_ (KTO) exhibited a stable cubic perovskite structure with a lattice constant of 4.005 Å. The MAPbBr_3_ (a = 5.98 Å) is approximate to 1.5 times that of KTO, which is similar to the condition of the CsPbBr_3_ and STO. Therefore, KTO was considered the epitaxial substrate for MAPbBr_3_. Fig. S9a shows the MAPbBr_3_ MPs array fabricated on the KTO substrate. Well-aligned of each pixel edges demonstrated the epaxial growth of MAPbBr_3_ on KTO. The rotation angles were concentrated into a narrow range from -2° to 4° (Fig. S9b). These results confirm the capability of the SC-ASC method to control the in-plane rotation of the perovskite array by combining the epitaxial growth strategy. To evaluate the crystal quality, the defect density and carrier mobility of the MPs were investigated. The trap density and carrier mobility were calculated to be 1.05 × 10^12^ cm^−3^ and 111.4 cm^2^ V^−1^ s^−1^, respectively (Fig. S10). The comparison of the trap density of the perovskite single-crystal array is displayed in Table S1. The above results reveal that the controllable prepared perovskite single crystal arrays by the SC-ASC method possess high crystal quality.

### Multicolor single-crystalline perovskite MPs libraries

**Fig. 3:**
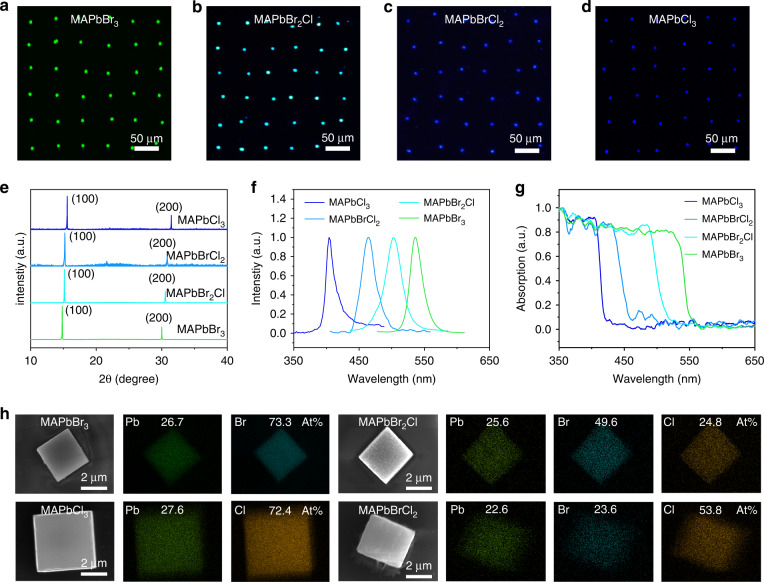
Multicolor single-crystalline perovskite MPs array characterization. Fluorescence micrograph of **a** MAPbBr_3_, **b** MAPbBr_2_Cl, **c** MAPbBrCl_2,_ and **d** MAPbCl_3_ MPs array under 365 nm light illumination. **e** XRD, **f** PL spectrum, and **g** absorption spectrum of the MPs array of MAPbBr_3_ (green), MAPbBr_2_Cl (cyan), MAPbBrCl_2_ (light blue), and MAPbCl_3_ (dark blue), respectively. **h** SEM images and EDS mapping results of MAPbBr_3_, MAPbBr_2_Cl, MAPbBrCl_2_, and MAPbCl_3_ MPs

Through mixing the precursors of MAPbBr_3_ and MAPbCl_3_ with certain ratios, a single-crystalline perovskite MPs array with different halogen element ratios can be obtained via this composition engineering. Due to the low solubility of MAPbCl_3_ in DMF, the mixture of DMSO/DMF with a ratio of 1:1 was employed as the solvent. Figure 3a–d demonstrates the fluorescence images of MAPbBr_3_, MAPbBr_2_Cl, MAPbBrCl_2_, and MAPbCl_3_ single crystal arrays excited under UV illumination with the wavelength of 365 nm, respectively. With the chlorine ratio increasing, the emission color is gradually transformed from bright green to dark blue. XRD analysis confirmed the structures of the as-fabricated perovskite arrays. As shown in Fig. 3e, two strong diffraction peaks, which are ascribed to (100) and (200) planes, have been observed for the four types of perovskite MPs, indicating the cubic structure. The PL spectra of these perovskite MPs array confirm the color changes (Fig. 3f). The wavelength of the PL peak of the MAPbBr_3-*x*_Cl_*x*_ (*x* = 0, 1, 2, 3) is centered at 542, 502, 464, and 410 nm, respectively. Figure 3g illustrates the absorption spectrum of these four perovskite arrays, showing the increase of the perovskite bandgap from 2.31 to 3.0 eV with Cl substituting for Br. The chemical composition and element distribution were analyzed by energy-dispersive X-ray spectroscopy (EDS), as shown in Fig. 3h. All the elements were homogeneously distributed in the entire crystals with the ratio approximate to the ideal stoichiometric of each perovskite. For example, the Pb, Br, and Cl were distributed uniformly in the MAPbBr_2_Cl crystals, showing the ratio of 1:1.9375:0.9688 which is closely around the ideal stoichiometry of 1:2:1. These capabilities of the SC-ASC strategy to regulate the fabrication process of the perovskite single-crystal arrays, including the pixel position, resolution, in-plane rotation, composition, confirm its potential application for large-scale integrated electronics and optoelectronics.

### Lasing behavior of the single-crystalline perovskite MPs array

**Fig. 4:**
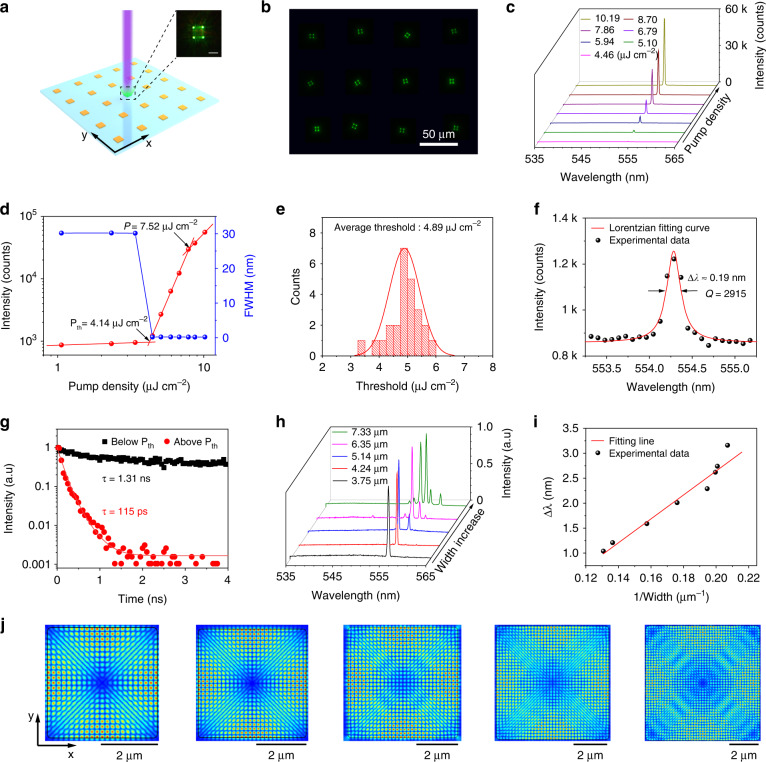
Lasing performance of the MAPbBr3 MPs array. **a** Schematic illustration of an individual MAPbBr_3_ MP excited by a 395 nm laser. The inset is a dark field image of a lasing MAPbBr_3_ MP. **b** Fluorescence microscopy image of MAPbBr_3_ MPs array excited by a 395 nm laser above the lasing threshold. **c** PL spectra of the as-synthesized perovskite MP pumped by a 395 nm laser with different densities. **d** Integrated PL emission intensity (red) and FWHM (blue) of PL peaks versus the pump density. **e** Statistics of the lasing threshold of MAPbBr_3_ MPs array. **f** Lorentzian fitting result of a lasing peak. **g** PL decay curves of the MAPbBr_3_ MPs obtained below and above the P_th_. **h** PL spectra of MAPbBr_3_ MPs with five different widths. **i** Δλ as a function of the MAPbBr_3_ MP width. **j** FDTD simulation of the two-dimensional electric field distribution inside the MAPbBr_3_ MPs labeled in (**h**)

The lasing resonance of the single-crystalline MAPbBr_3_ MPs array has been carefully investigated. A customized optical measurement system consisting of a confocal microscope, a femtosecond laser source (395 nm), and a spectrometer was employed to characterize the laser performance of the MAPbBr_3_ MPs array (Fig. S11). Figure 4a schematic illustrates the lasing process of an individual pixel. When the pump density is lower than the lasing threshold (P_th_), the MAPbBr_3_ MP demonstrated a broad spontaneous emission with fluorescence emission from the entire crystal. With pump density increasing to exceed the P_th_, four bright emissive corners of the square MPs were observed (the inset of Fig. 4a). Figure 4b shows the dark field image of a 4 × 3 MAPbBr_3_ MPs array excited by a 395 nm laser above P_th_. All the MPs exhibited four bright spots at the corner. The PL spectra of the perovskite MPs array with a width of 3 μm under different densities confirm the lasing behavior. As shown in Fig. 4c, when the pump density increased to 4.14 μJ cm^−2^, a narrow and sharp peak appeared and increased rapidly with the pump density. With continuously increasing the pump density, only one lasing peak was observed, indicating the behavior of a single-mode laser. The plot of the integrated PL intensity in the log-log scale with increasing pump density and full-width at half-maximum (FWHM) as a function of the pump density are illustrated in Fig. 4d. An “S”-shaped curve of the integrated PL intensity was observed, which refers to the light-in-light-out (L-L) relation of the lasing oscillation. The P_th_ derived from the “S” curve is 4.14 μJ cm^−2^ and the threshold for lasing oscillation is 7.52 μJ cm^−2^. At the density of P_th_, the FWHM demonstrates a dramatic decrease. The P_th_ of 25 perovskite MPs randomly distributed in the array was summarized in Fig. 4e. Over 80% of MPs exhibited a low P_th_ of 4.25 to 5.75 μJ cm^−2^, verifying the uniformity of the as-fabricated perovskite single crystal array. Figure 4f demonstrates the PL spectrum pumped with the intensity of 1.08P_th_ (4.46 μJ cm^−2^), where the FWHM was extracted to be 0.19 nm by fitting this curve with the Lorentz function. The quality factor (Q), defined as Q = λ/FWHM, was calculated to be ~2915. The typical PL decay curves of the MAPbBr_3_ MP obtained below and above the P_th_ were demonstrated in Fig. 4g. Through fitting the decaying profiles with an exponential function, the lifetimes were extracted. The fast component and slow component of the lifetime are calculated to be 1.41 and 35.9 ns (below P_th_), respectively (Fig. S12). With increasing the excitation intensity, the lifetime drops to 115 ps (above P_th_) which can be attributed to the appearance of the stimulated emission process. We compared the lasing behavior of perovskite arrays fabricated through our SC-ASC method with the representative perovskite lasers (Table S2). There is a gap compared with the state-of-art perovskite laser fabricated through the vapor method, however, considering the readily control of the individual pixels, it can be enhanced by utilizing the high-crystalline MPs array with further control of the crystallization process. Finite-difference time-domain (FDTD) simulations were utilized to identify the dominant resonant cavity of MAPbBr_3_ MPs (Fig. S13). The dominant oscillation modes for optical wave propagation in the perovskite microcavity usually consist of the transverse magnetic (TM) mode and the transverse electric (TE) mode, however, only the one mode that possesses high-quality factor could realize the resonance. The WGM microcavity in TM mode exhibited a stronger restraint effect for optical waves and allowed total reflection between the four internal sidewalls, while there are no contributions from the TE waves for the lasing behavior (Fig. S14). The Fabry–Pérot (F-P) mode between the top and bottom surface demonstrates a small quality factor, which is absent in the MAPbBr_3_ MPs.

The dimensions of MAPbBr_3_ MPs determine the optical wave propagation models inside the crystals and therefore yield diverse lasing outputs. Since the dimensions of the as-fabricated MPs can be easily controlled by hydrophilic areas in our strategy, the single-mode, dual-mode, and multimode lasing have been realized. Figure 4h illustrates the PL spectra of the MAPbBr_3_ MPs with different widths. With the width increasing, the laser is transited from single to multimode lasing. For the square perovskite WGM microcavity, the mode spacing (Δλ) can be expressed by the equation:

1 $$\Delta \lambda = \frac{{\lambda ^2}}{{L(n - \lambda dn/d\lambda )}}$$

where *λ* is the emission wavelength, (*n - λdn/dλ*) is group refractive index^45^ and *L* is the cavity length of the WGM cavity calculated by $L = 2\sqrt 2 \,W$ (*W* is the width of square perovskite MP), which represented the path of light wave in the WGM cavity for one cycle. According to Eq. 1, the linear relationship between Δλ and 1/width further confirms the WGM microcavity of the MAPbBr_3_ MPs (Fig. 4i). FDTD simulation was then carried out to reveal the fundamental lasing mode in the MAPbBr_3_ MPs. The dimensions of the MPs in Fig. 4h were adopted in the simulation. Figure 4j exhibits the electric field distribution of TM mode. With the increasing width of perovskite MPs, the number of electric field layers inside the microcavity increases, which is attributed to the enhanced confinement effect of the electric field. Thus, the number of the laser mode increases with the width of the microcavity, which is consistent with the results of the lasing behavior in Fig. 4h.

### Demonstration of the perovskite photodetector array

**Fig. 5:**
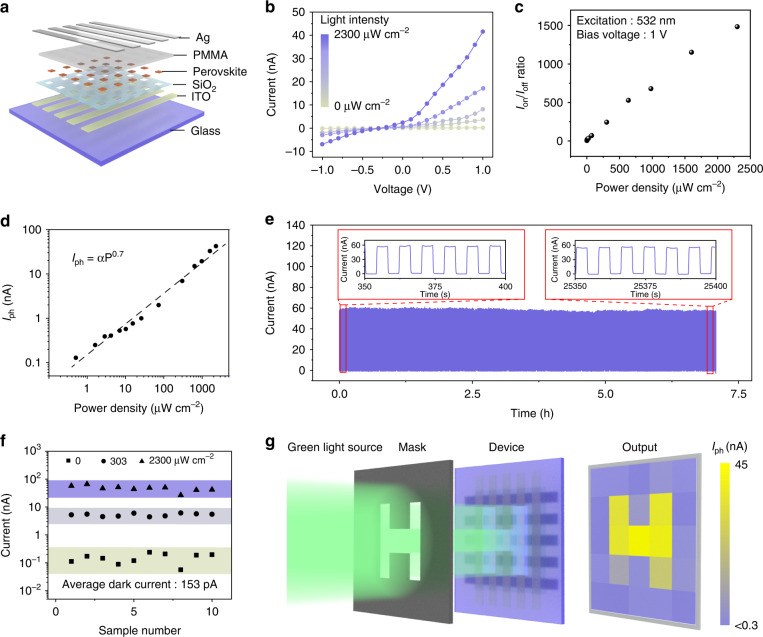
Vertically structured photodetector array based on the MAPbBr3 MPs. **a** Schematic illustration of the device structure of the photodetector array. **b** *I-V* curves of the device measured under the 532 nm illumination with light intensity from 0 to 2300 μW cm^−2^. **c** I_on_/I_off_ ratio as a function of the light intensity. **d** The dependence of I_ph_ on the light intensity. **e** Stability of the device after continuous operation for 7 h. **f** Dark current and photocurrent of 10 pixels in a photodetector array. **g** Light intensity distribution imaging through mapping the photocurrent of the photodetector array

Using the SC-ASC method, the perovskite MPs array can be easily synthesized on different substrates and aligned with prepared patterns. We proposed a perovskite photodetector array with vertical device architecture by generating the perovskite MPs on an electrode array. Figure 5a illustrates the structure of the photodetector array, which consists of the ITO strips as the bottom electrodes, a patterned perovskite array as the active layer, a thin layer of polymethyl methacrylate (PMMA) for insulation and the Ag stripes orthogonal to the ITO stripes as the top electrodes. To increase the photocurrent of the device, each pixel contains a 20 × 20 aligned single-crystalline MAPbBr_3_ MPs array. The fabrication process of the perovskite photodetector array is schematically illustrated in Fig. S15 and the detailed fabrication process can be found in the Supplementary Materials. Figure 5b depicts the current-voltage (*I-V*) curves of a single pixel under the green light illumination (532 nm) with different light intensities varying from 0 to 2300 μW cm^−2^. With light intensity increasing, the current increases owing to the generation of a large number of photogenerated carriers. The on/off current ratio (*I*_on_/*I*_off_) as a function of light intensity was plotted in Fig. 5c. *I*_on_/*I*_off_ ratio of 1484 was obtained under the illumination intensity of 2300 μW cm^−2^. The response time and the decay time of the perovskite photodetector with electrodes prepared by shadow mask directly were measured to be 0.5 and 0.78 ms, respectively (Fig. S16). The photocurrent (*I*_ph_) versus the illumination intensity was plotted in Fig. 5d (black dashed line), which can be fitted by the equation:

2 $$I_{{{{\mathrm{ph}}}}} = \alpha P^\beta$$

where *I*_ph_ is the photocurrent defined as *I*_ph_ = *I*_light_ *- I*_dark_, *P* is the light intensity, *α* and *β* are constants which are both the fitting parameters. The *β* is fitted to be 0.7, which indicates a sublinear power-law behavior. The sublinear relationship is caused by the complex process of electron-hole generation, recombination, and trapping with a semiconductor material^46^. The linear dynamic range (LDR) was calculated to be 50.35 dB (Fig. S17). The responsivity (R) is another important parameter of photodetectors and it can be expressed by the equation *R* = *I*_ph_*/PA*. The maximum responsivity of the device is 7 A W^−1^. Besides, to calculate the detectivity of the device, the NEP of the MAPbBr_3_ photodetector is calculated to be 1.43 × 10^−14^ W Hz^−1/2^ at a modulation frequency of 1 Hz (Fig. S18), showing a maximum detectivity of 4.2 × 10^11^ Jones that was calculated with the responsivity of 7 A W^−1^ (see the Supplementary Materials). The photodetector array demonstrated stable dynamic photoswitching behavior. As shown in Fig. 5e, no obvious decaying of the photocurrent can be observed after the device was working continuously for 7 h.

To demonstrate the application of the perovskite photodetector for the imaging of perceived patterns, we first verified the uniformity of output current from ten pixels. Although the dark currents varied from 56 to 240 pA, the photocurrents under both two illumination intensities were distributed in a narrow range (Fig. 5f). Subsequently, an “H” shaped shadow mask was adopted to generate patterned illumination on the perovskite photodetector array for light imaging. The output current of each pixel was collected by connecting the corresponding top and bottom electrodes (Fig. S19). Through mapping the output current from each pixel, a clear letter of “H” can be observed, showing the reliable imaging function of the perovskite photodetector array (Fig. 5g).

## Discussion

We have demonstrated a facile one-step SC-ASC strategy for scalable growth of single-crystalline perovskite MPs array on various substrates. This fabrication method enables precise control of the array’s properties, including the shape and resolution of the array and the dimensions and in-plane rotations of individual pixels. Due to the high quality of the perovskite crystals, systematical dimension-dependent laser emission is explored and both the laser mode and emission wavelength are effectively modulated by crystal size. In addition, based on the fabrication of the perovskite array aligned on the patterned electrodes, an independently addressable photodetector array with a vertical structure has been demonstrated, realizing the imaging of input patterns. The concept of this strategy is universal and we expect that it can be expanded to fabricate other solution-processed semiconductors.

## Materials and methods

### Fabrication of pre-patterned substrate

The target substrate with desired dimensions was firstly cleaned with acetone, alcohol, and deionized water followed by the oxygen plasma treatment. Then, the photoresist patterns were generated on the substrate through traditional photolithography. Subsequently, a thin film of SiO_2_ was prepared through radio frequency (RF) magnetron sputtering (Kurt J. Lesker PVD75). Then, the substrate was soaked in a mixed solution of n-hexane and octadecyl trichlorosilane (OTS) with a ratio of 300:1 for 15 min followed by the photoresist stripping in acetone for 5 min. Finally, the as-prepared substrate was cleaned with deionized (DI) water and dried with nitrogen.

### Fully-controlled growth of perovskite single-crystal arrays

For the fabrication of the MAPbBr_*x*_Cl_3-*x*_ (*x* = 0–3) MPs array, two solutions were first prepared. One was prepared by dissolving the methylammonium bromide (MABr) and lead bromide (PbBr_2_) in the DMF solution with a molar ratio of 1:1. Another one was prepared by using a mixture solution of DMF and DMSO (1:1) to dissolve the methylammonium chloride (MACl) and lead chloride (PbCl_2_). The two prepared solutions were mixed with the ratio of 1:0, 1:2, 2:1, and 0:1 to serve as the perovskite precursor solution with a concentration of 0.6 M. Then, the precursor solutions were drop-casted on the target substrate, leaving the liquid in the hydrophilic areas. Subsequently, a blank glass substrate with a hydrophobic surface was placed on the target substrate to form space confinement with a clamp to hold the two substrates. The gap between the hydrophobic glass and the target substrate can be tuned from 9 to 45 μm by changing the pressure of the clamp. Then, the antisolvent-assist crystallization was performed at 50 °C in the CH_2_Cl_2_ atmosphere. During the crystallization process, the relative position between the target substrate and confinement substrate was tuned to make sure that the pixels are well-aligned. Then, after the solvent has evaporated, the peeling off of the confinement substrate completed the fabrication process of the perovskite array.

### Preparation of perovskite single-crystal arrays on STO and KTO

Firstly, the STO substrate was cleaned with DI water at 50 °C for 30 min and further treated with 20% HF at room temperature for 2 min to generate the Ti-O terminated surface. Secondly, for the STO substrate, the patterned substrate with CsPbBr_3_ precursor domains array (0.3 M) was confined by the as-prepared STO substrate instead of the hydrophobic glass. Then, the perovskite crystals were synthesized in the antisolvent atmosphere for 12 h. After peeling off the glass substrate, the perovskite crystals were located at the STO substrate. KTO substrate was treated similarly to the treatment of the STO substrate. Then, MAPbBr_3_ (0.6 M) precursor was applied on the patterned substrate and covered the KTO substrate. After growth at 50 °C for 6 h, the patterned substrate and KTO were separated and the perovskite crystals were located at the KTO substrate.

### Characterization of single crystal arrays

An optical microscope (Zeiss Observer Z1) and a scanning electron microscope (Hitachi SU8020) were used to characterize the morphologies of the as-synthesized perovskite arrays. The structure characterization was performed by a transmission electron microscope (JEM-2010). The phase analysis was carried out by X-ray diffraction (X’Pert3 Powder). Fluorescence images in the dark field were recorded by a metallurgical microscope with the excitation of a 365 nm laser. The steady-state/transient fluorescence spectrometer (FLS980) was adopted for the PL spectra measurements. The absorption spectra of samples were measured by a UV-Vis-NIR spectrophotometer (Shimadzu UV 3600).

### Lasing measurement

The light source for perovskite lasing measurements was the femtosecond laser (Stimulated light with 395 nm, frequency 1000 Hz) coupled with a confocal μ-PL system (Zeiss M1). The emitted light was collected by a CCD detector joint with an optical multichannel analyzer (Andor, SR-500i-D1-R). The PL decay curves were measured by a photon counting detector (PicoQuant, PDM series) with a resolution of ~ 100 ps.

### Fabrication of photodetector array and device characterization

ITO electrode arrays (thickness ~100 nm) were first deposited by RF magnetron sputtering. Then, the patterned hydrophilic-hydrophobic arrays were fabricated by the method shown in Fig. S1. Subsequently, the PMMA solution prepared by dissolving PMMA particles in chlorobenzene (100 mg ml^−1^) was spin-coated on the as-synthesized perovskite single crystal arrays at 1000 r min^−1^. Next, the sample was treated with reactive ion etching (RIE) (PDC100B Plasma Cleaner) at high power (~160 W) with 50 sccm O_2_ and 50 sccm Ar for 6 min. Finally, the top Ag electrode arrays were deposited by RF magnetron sputtering with a shadow mask. The current of the photodetectors was measured with the Keithley 4200 and Stanford SR570. The light source of a 532 nm laser was adopted for investigating the photoresponse properties. A customized multichannel system was employed to characterize the imaging capabilities.

## Supplementary information

Supplementary Information for Controlled On-chip Fabrication of Large-scale Perovskite Single Crystal Arrays for High-performance Laser and Photodetector Integration

## Data Availability

All data needed to evaluate the conclusions in the paper are present in the paper and/or the Supplementary Materials. Additional data related to this paper may be requested from the authors.
