# Supplementary material for: Controlled on-chip fabrication of large-scale perovskite single crystal arrays for high-performance laser and photodetector integration
Source: Light Sci Appl. 2023 Mar 8;12:67. doi: 10.1038/s41377-023-01107-4 (PMC9992671; doi:10.1038/s41377-023-01107-4)
Supplement: Supplementary file 1 — Supplementary Information for Controlled On-chip Fabrication of Large-scale Perovskite Single Crystal Arrays for High-performance Laser and Photodetector Integration [file 41377_2023_1107_MOESM1_ESM.docx]

Supplementary Information for

**Controlled On-chip Fabrication of Large-scale Perovskite Single Crystal Arrays for High-performance Laser and Photodetector Integration**

Zhangsheng Xu^1, 2^, Xun Han^1, 3*^, Wenqiang Wu^1, 4^, Fangtao Li^1^, Ru Wang^1 ,4^, Hui Lu^1, 2^, Qiuchun Lu^1, 5^, Binghui Ge^6^, Ningyan Cheng^6^, Xiaoyi Li^7^, Guangjie Yao^8^, Hao Hong^8^, Kaihui Liu^8,9^ and Caofeng Pan^1,2 *^

^1^CAS Center for Excellence in Nanoscience, Beijing Key Laboratory of Micro-nano Energy and Sensor, Beijing Institute of Nanoenergy and Nanosystems, Chinese Academy of Sciences, Beijing, 101400, China

^2^School of Nanoscience and Technology, University of Chinese Academy of Sciences, Beijing, 100049, China

^3^College of Mechatronics and Control Engineering, Shenzhen University, Shenzhen, 518060, China

^4^College of Physics and Optoelectronic Engineering, Shenzhen University, Shenzhen, 518060, China

^5^Center on Nanoenergy Research, School of Physical Science and Technology, Guangxi University, Nanning, 530004, China

^6^Information Materials and Intelligent Sensing Laboratory of Anhui Province, Key Laboratory of Structure and Functional Regulation of Hybrid Materials of Ministry of Education, Institutes of Physical Science and Information Technology, Anhui University, Hefei, 230601, China

^7^College of Materials Science and Engineering, Ocean University of China, Qingdao, 266100, China

^8^State Key Laboratory for Mesoscopic Physics, Frontiers Science Centre for Nano-optoelectronics, School of Physics, Peking University, Beijing, 100871, China

^9^Songshan Lake Materials Laboratory, Dongguan, 523808, China

These authors contributed equally: Zhangsheng Xu, Xun Han, Wenqiang Wu.

^*^Corresponding author. Email: Xun Han ([hanxun@szu.edu.cn](mailto:hanxun@szu.edu.cn); tel:+8618810869039 ); Caofeng Pan ([cfpan@binn.cas.cn](mailto:cfpan@binn.cas.cn); tel:+8618518768163)

Supplementary Text

**Discussion about the influence of the gap on the thickness of perovskite.**

The gap can be tuned by changing the pressure of the clamp. After placing the hydrophobic glass substrate on the target substrate, we use a clamp to hold the two pieces of substrate. By changing the pressure of the clamp, the gap could be tuned from 9 μm to 45 μm.

As shown in Fig. S2a, the thickness of the MPs decreases with reducing the gap between two substrates while the thickness of MPs remains smaller than the gap. However, the crystals fabricated with the gap of 9 μm demonstrate irregular shapes, which is because the perovskite tends to nucleate at the corner of the hydrophilic squares and grow along the edges (Fig. S2b). Under the large gap conditions, the edges of crystals become smooth due to the uncontrolled evaporation rate of the solvent of the precursor solution (Fig. S2c). Considering the control of the crystallization process and pixel positions, we adopted the gap of 20 μm in this work for the fabrication of perovskite crystal arrays.

**Calculation of the trap density and carrier mobility.**

For easy measurement, a large hydrophilic window is utilized for the crystal array growth. We firstly measured the I-V curve of the MAPbBr_3_ crystals in the dark condition (Fig. S9). It shows three different regimes: the ohmic regime at low bias, the trap-filled regime with a starting point of V_TFL_, and the child regime at high bias. The trap density (n_trap_) and carrier mobility (μ) could be calculated by the following equations ^1, 2^:

 (1)

 (2)

where *ε* is the relative dielectric constant, ε_0_ is the vacuum permittivity, L is the distance between two electrodes, J_D_ is the current density at the applied voltage of V_b_. As a consequence, the trap density and carrier mobility were calculated to be 1.05 ×10^12^ cm^-3^ and 111.4 cm^2^ V^-1^ s^-1^, respectively.

**Discussion of the perovskite MP cavity.**

To confirm that the main contribution to the laser is the whispering gallery mode (WGM) cavity rather than Fabry-Pérot (F-P) cavity (Fig. S9), the quality factor (Q factor) of the F-P cavity is calculated by the equation:

$Q=\frac{2\pi n_{1}L}{\lambda(1-R)}$ (3)

where *n_1_* is the refrae of the perovskite MP, *L* is the cavity length and *λ* is the emission wavelength and *R* is the reflectivity of the boundary. *R* can be calculated according to the equation:

$R=[\frac{n_{1}\cos\theta_{1}-n_{2}\cos\theta_{2}}{n_{1}\cos\theta_{1}+n_{2}\cos\theta_{2}}]^{2}$ (4)

where *n*_2_ is the refractive index of air, *θ*_1_ and *θ*_2_ are the angle of incidence and refraction which can be considered as 0° in the F-P cavity. In addition, the stimulated radiation wavelength and the thickness of the perovskite MP was measured in the experiment. According to the above formula, we can calculate Q factor of the F-P cavity as 92.6, which is much smaller than the test results (Q factor = 2915). Hence, the F-P cavity hardly contributes to the stimulated emissions ^2-5^.

**The preparation process of the perovskite photodetector array.**

1. ITO electrodes were deposited on the glass by RF Magnetron sputtering (80 W, 30 min).
2. The patterned substrate was obtained by the preparation process (Fig. S1).
3. Perovskite MP array was fabricated according to the method as shown in Fig. 1a.
4. PMMA as insulating and protective layers was spin-coated on the perovskite MP array (1000 rpm s^-1^, 1 min) and cured at 80 ℃ for 5 min.
5. After finishing PMMA coverage, PMMA thin film was etched by RIE (~ 160 W, 1 min) for removal of redundant PMMA on the perovskite MP array.
6. Patterned Ag as top electrodes was deposited with PET mask (prepared by laser cutting machine) by RF magnetron sputtering (60 W, 3 min).

**Analysis of noise spectral density.**

The noise in the dark current of the photodetector array was analyzed by a reported method ^5^ using a power source, a low noise current preamplifier (Stanford Research System SR 570) and an oscilloscope (Keysight, InfiniiVision MSOX4154A). The dark current was measured with a sampling rate of ~ 645 Hz, as shown in Fig. S17a. Through a Fourier transform (FFT) of the measured dark current ^6^, the noise spectral density was obtained, as shown in Fig. S17b. Then, the noise-equivalent-power (NEP) can be calculated by dividing the noise spectral density by the responsivity. The NEP of the MAPbBr_3_ photodetector is calculated to be 1.43×10^-14^ W Hz^-1/2^ at a modulation frequency of 1 Hz. Thus, the specific detectivity (D*) could be calculated by

$D*=\frac{\sqrt{AB}}{NEP}$ (7)

where A is the device area. The D* of the MAPbBr_3_ photodetector is calculated to be 4.2×10^11^ Jones.


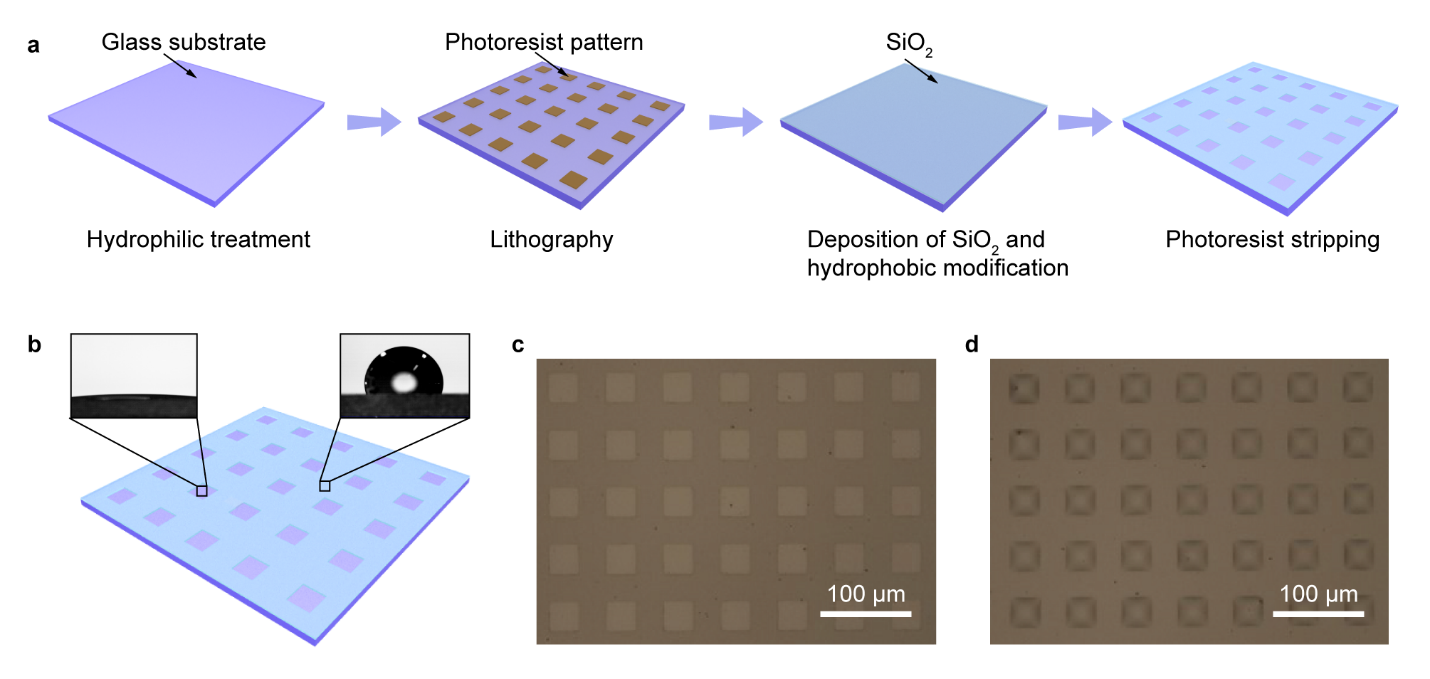


Fig. S1. a Schematic illustration of the substrate preparation process. b The image of contact angle in different areas. The optical image of the patterned substrate c before and d after smearing perovskite solutions


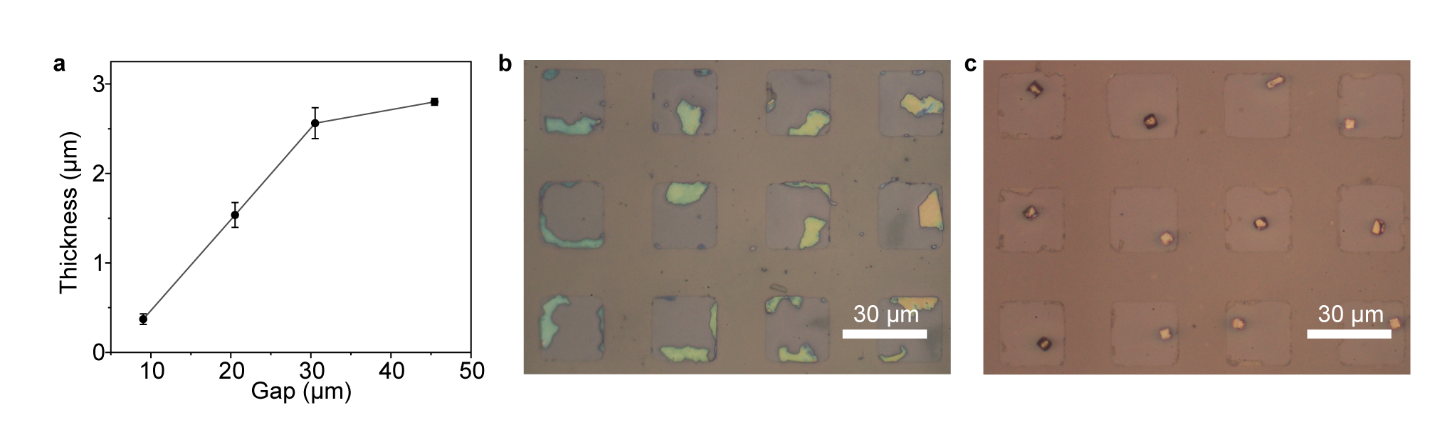


**Fig. S2.** **a** The dependence of thickness of MPs on the gap between target and confinement substrate. The optical images of the MPs synthesized under **b** small and **c** large gap conditions.


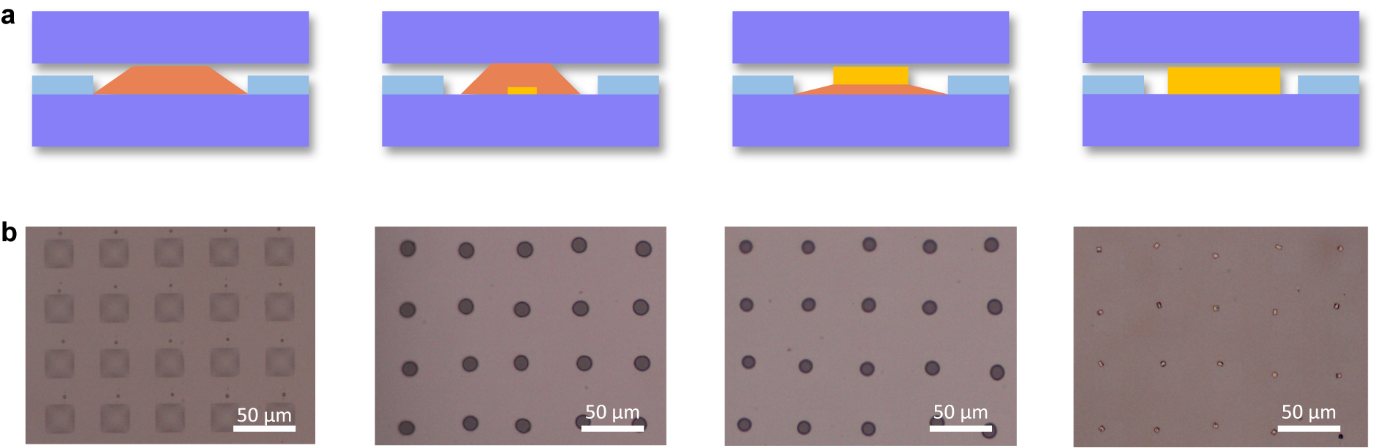


Fig. S3. a Schematic illustration of the growth process about a perovskite MP. b The top-view microscopical optical image of samples.


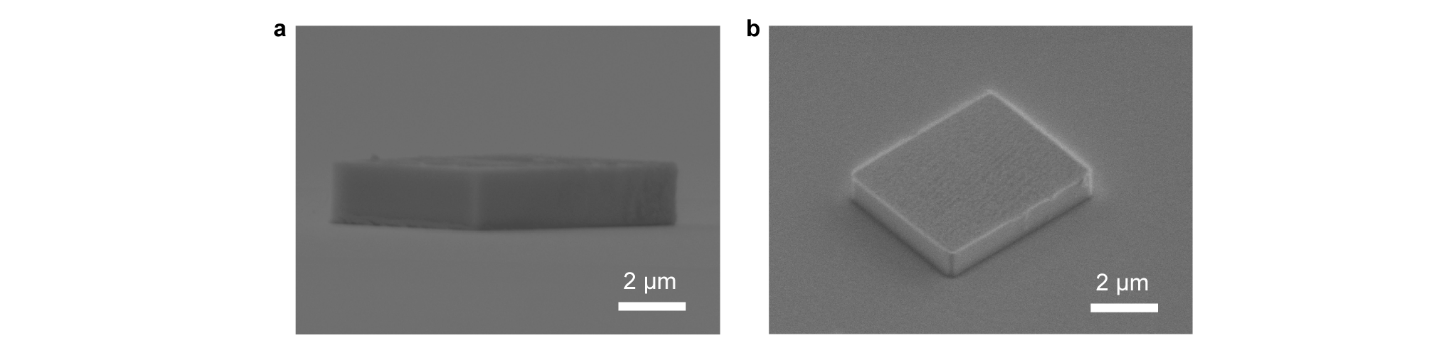


Fig. S4. SEM images of MAPbBr_3_ MP with different perspectives.


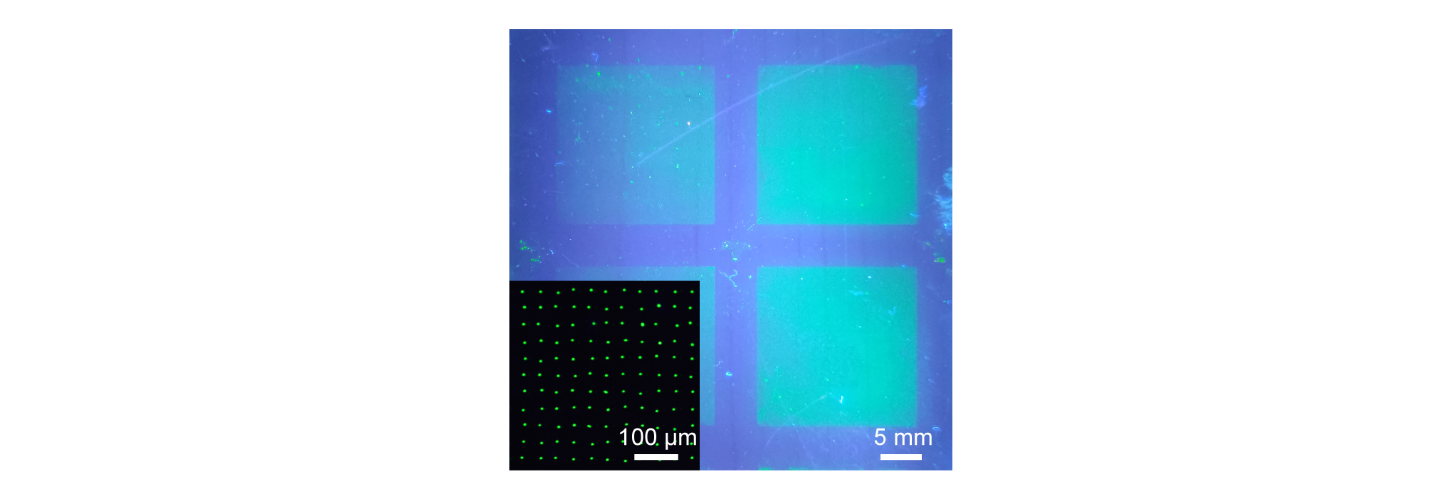


Fig. S5. Fluorescence image of the large-scale perovskite array excited by 365 nm illumination. The inset is an enlarged image.


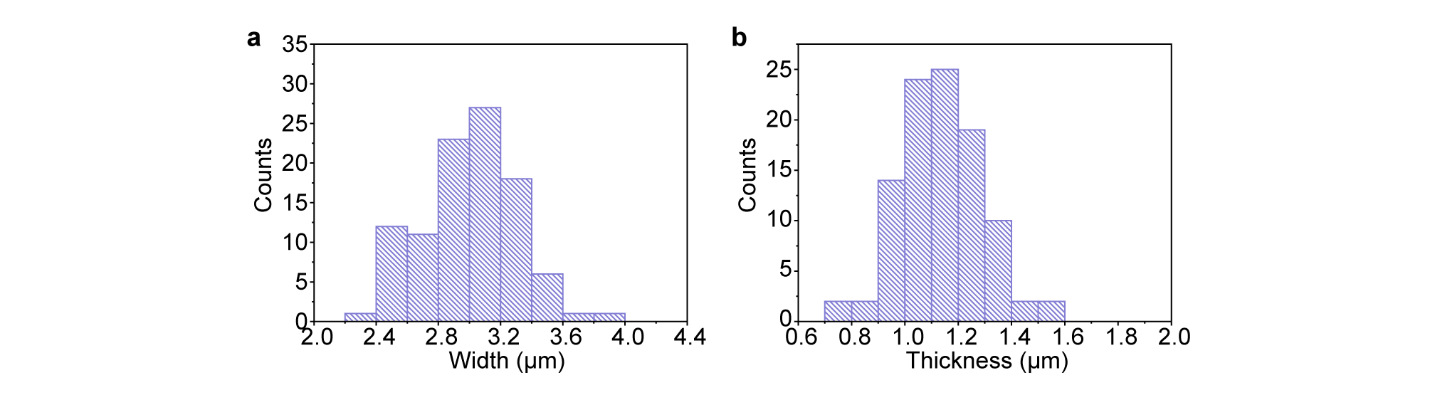


Fig. S6. Distribution of a width and b thickness of 100 perovskite MPs.


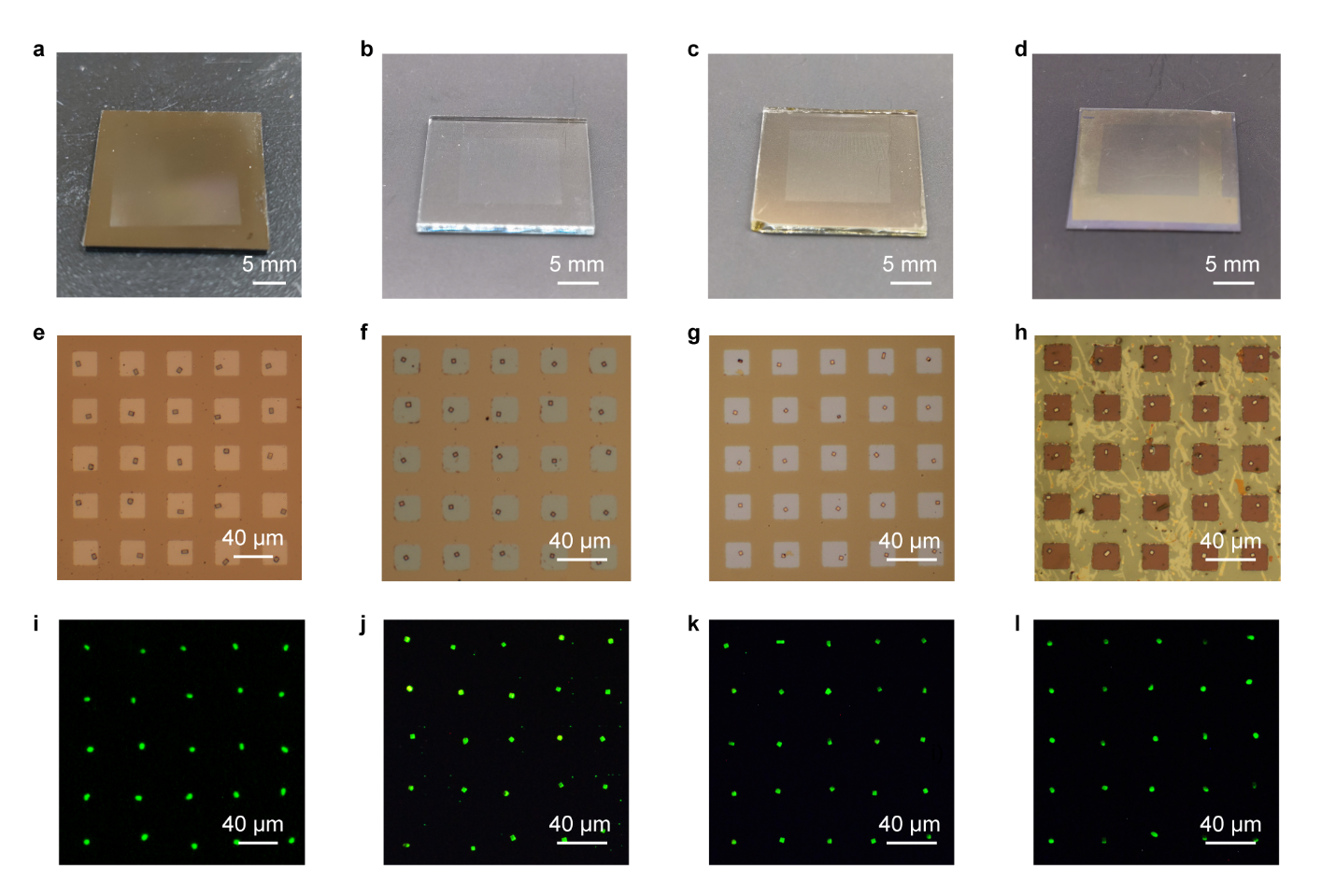


**Fig. S7**. Optical images of the as-fabricated perovskite MPs array on **a** Cr, **b** ITO, **c** NiO, and **d** PET substrate. **e-h** The corresponding microscope photograph of perovskite MP arrays. **i-l** Fluorescence image of the perovskite MPs array fabricated on different substrates.


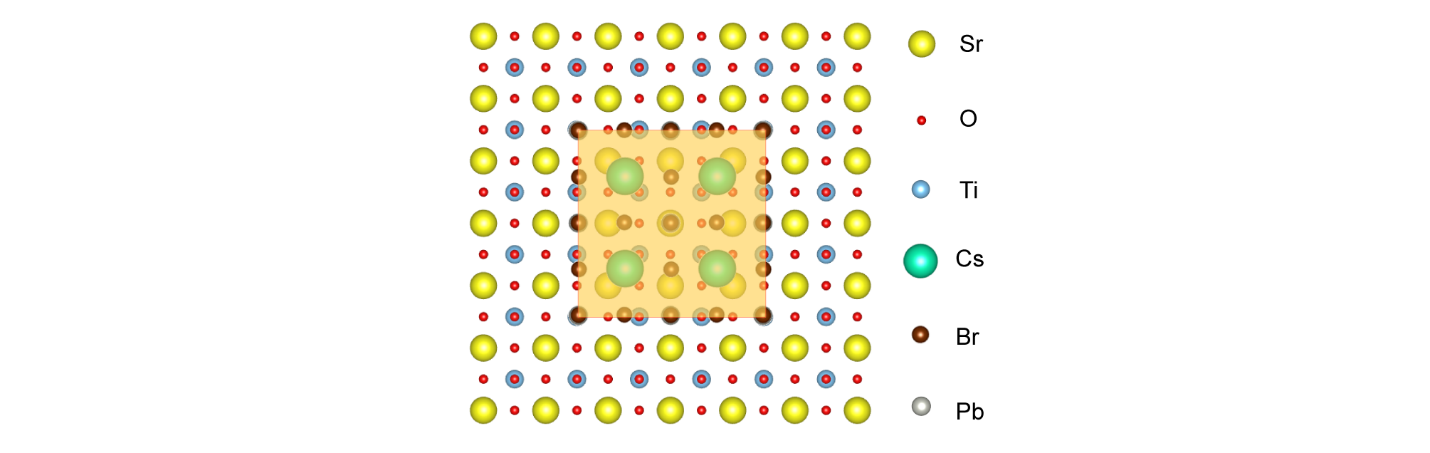


Fig. S8. Schematic illustration of lattice about CsPbBr_3_ on STO (100) substrate.


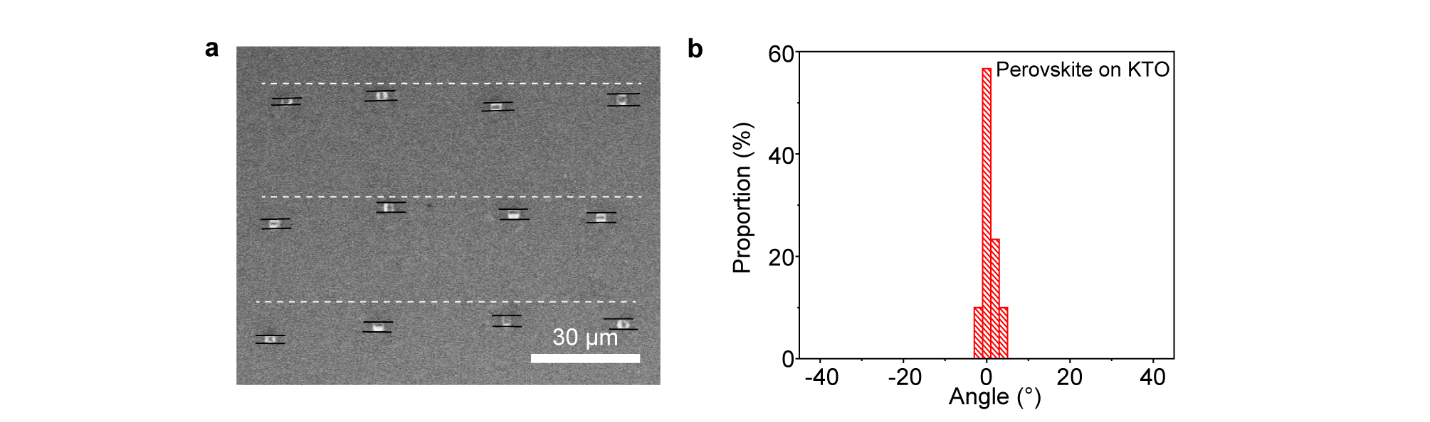


**Fig. S9.** **a** SEM image of MAPbBr_3_ MPs array synthesized on KTO (100) substrate. **b** Statistics of the in-plane rotation of 100 perovskite MPs array synthesized on KTO (100) substrate.


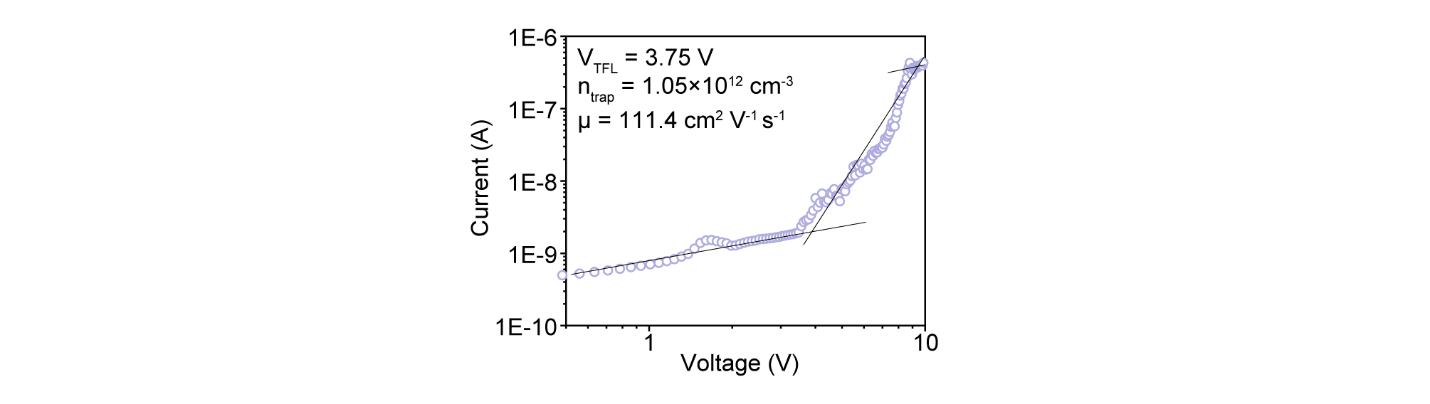


**Fig. S10**. I-V characterization of the MAPbBr_3_ MPs devices in the dark condition.


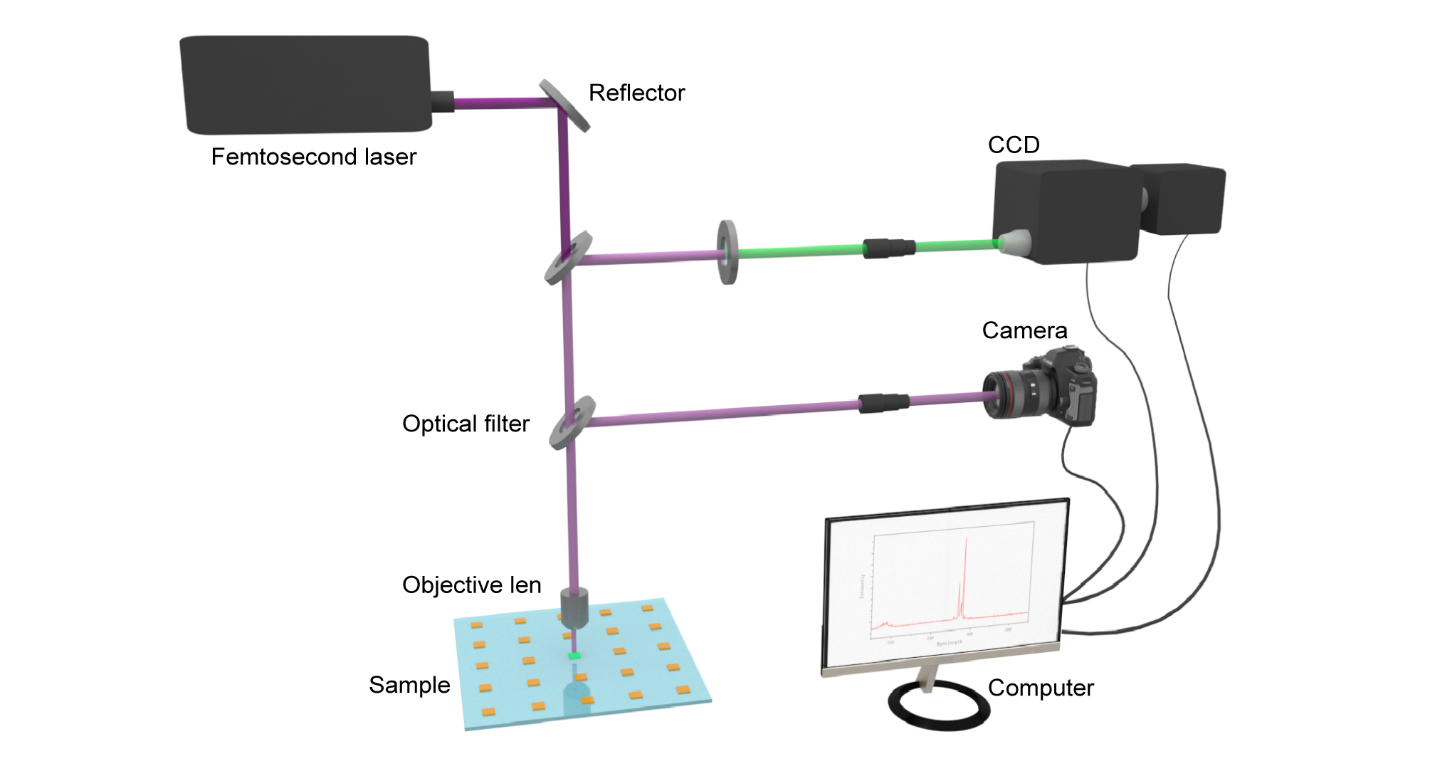


Fig. S11. Schematic illustration of the entire optical path and test system for laser measurement.


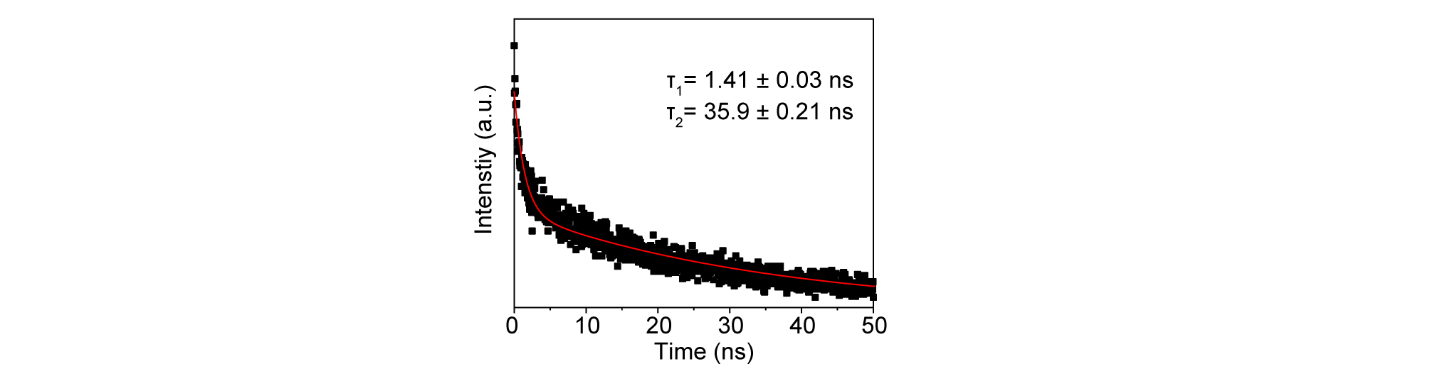


**Fig. S12.** PL decay trace of the MAPbBr_3_ MPs.


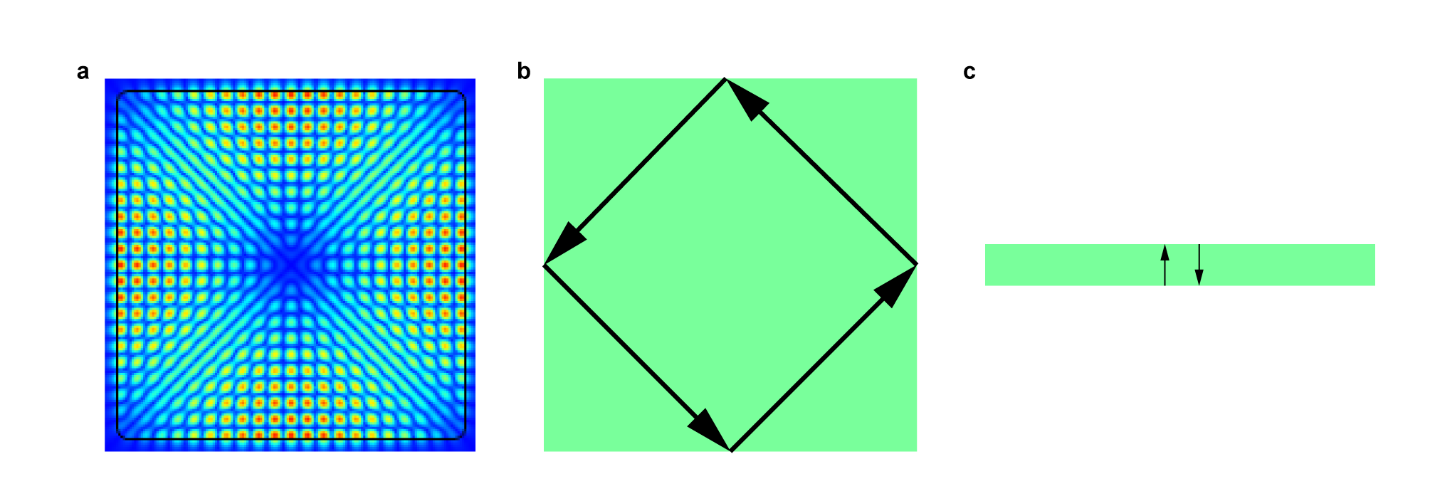


Fig. S13. a FDTD simulations of WGM mode. Schematic diagram of the lightwave path in b WGM cavity and c F-P cavity in the perovskite MP.


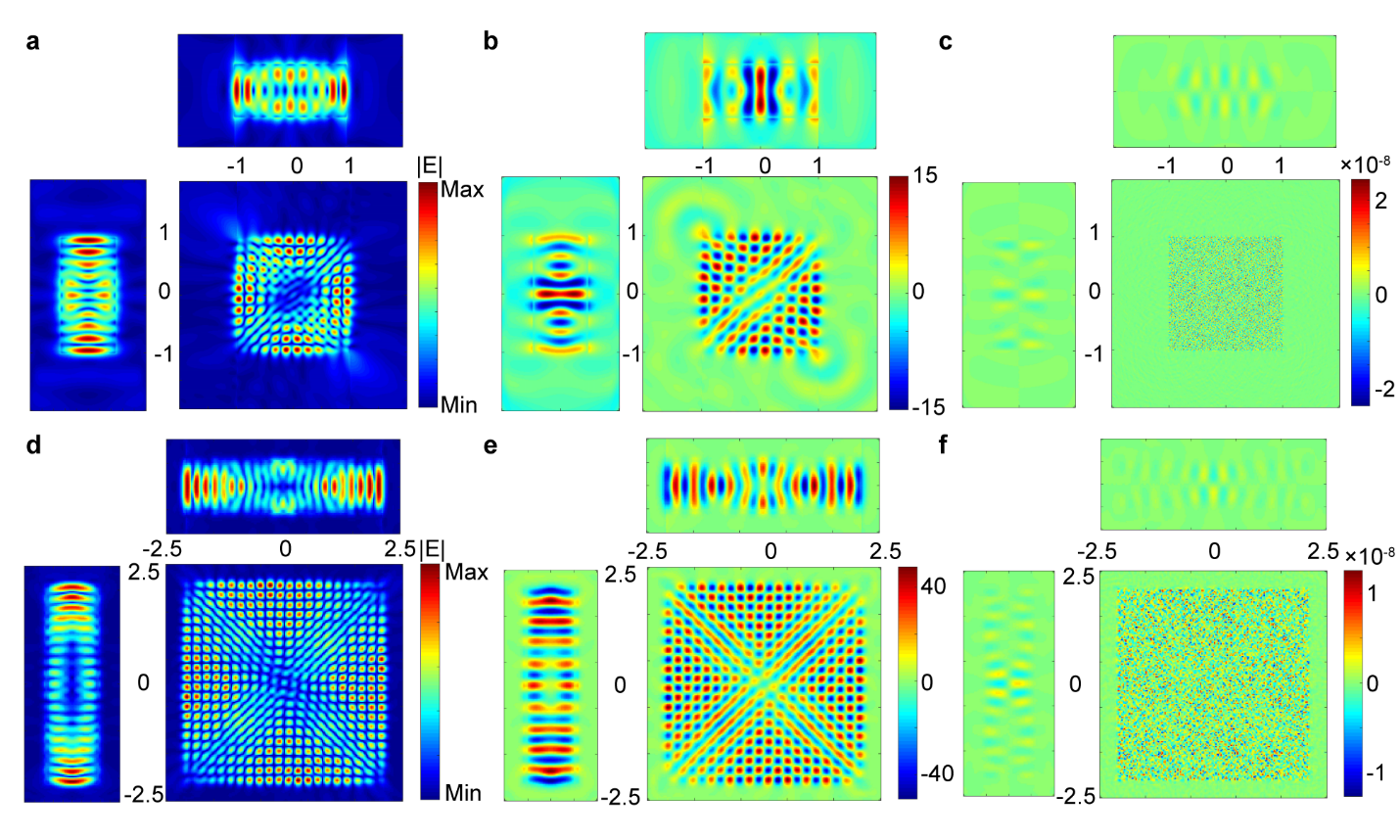


**Fig. S14**. **a** The absolute electric field distribution |E| pattern in a 2.0×2.0×0.6 μm^3^ square MP, presented by an optical mode nm with a cavity quality factor Q = 1003. **b** and **c** are electric and magnetic field distributions of this mode in the z-direction. **d** The absolute electric field distribution |E| pattern in a 3.75×3.75×0.9 μm^3^ square MP, presented by an optical mode with a cavity quality factor Q = 3695. **e** and **f** are electric and magnetic field distributions of this mode in the z-direction. As shown in **c** and **f**, the magnetic field distribution in the z-direction is negligible, thus this mode is assigned as a TM mode.


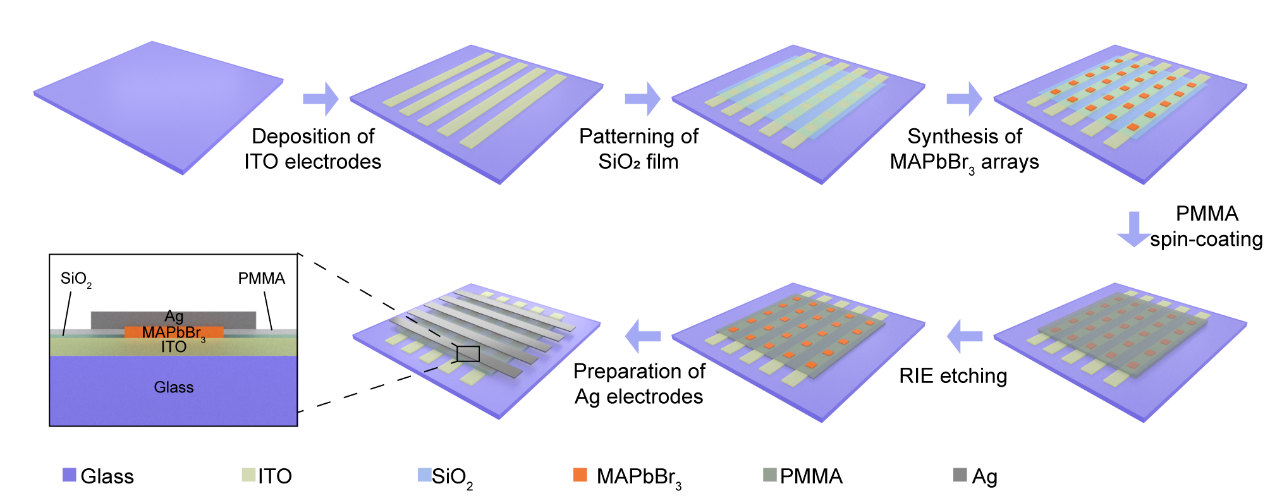


Fig. S15. Schematic illustration of the preparation process of the perovskite photodetector array.


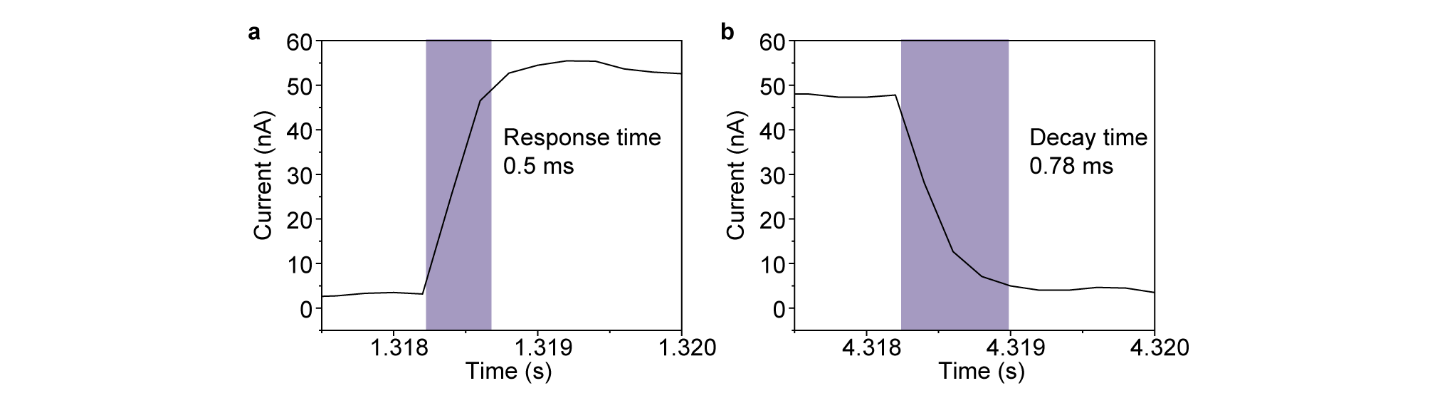


**Fig. S16.** Response time and decay time of the MAPbBr_3_ crystal photodetector.


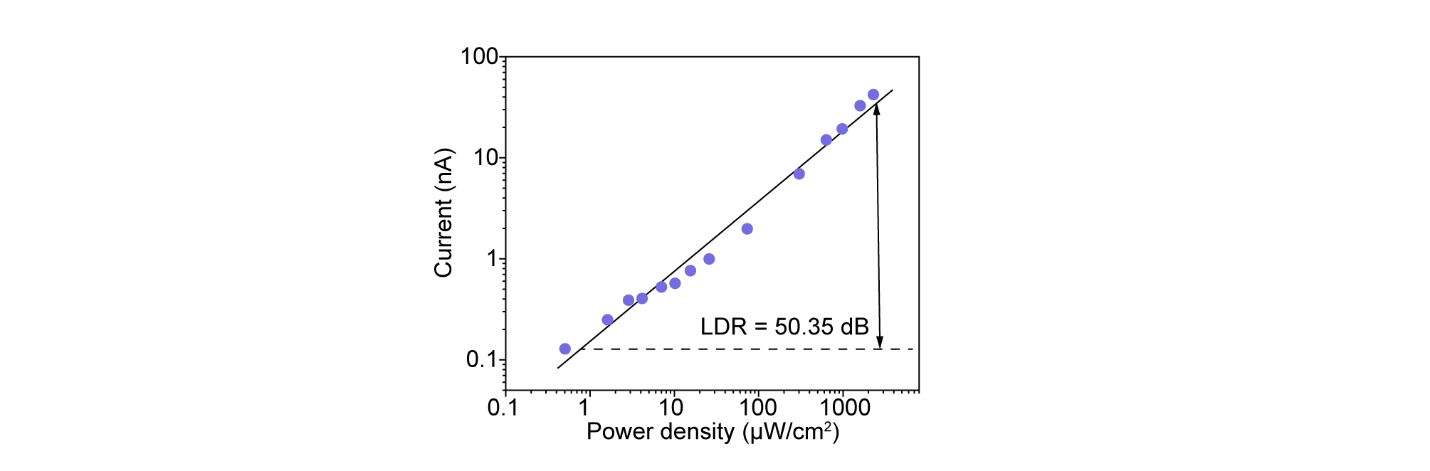


**Fig. S17**. The dependence of current on power density at a bias voltage of 1 V.


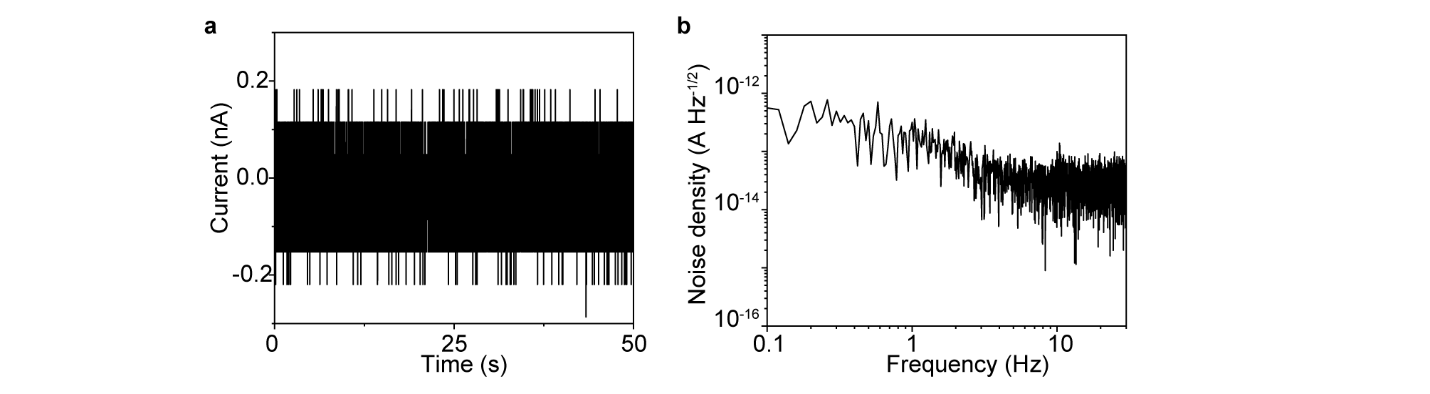


**Fig. S18**. **a** The dark current waveform of the MAPbBr_3_ photodetector arrays. **b** Analysis of noise spectral density of photodetector based on the dark current waveform measured in **a**.


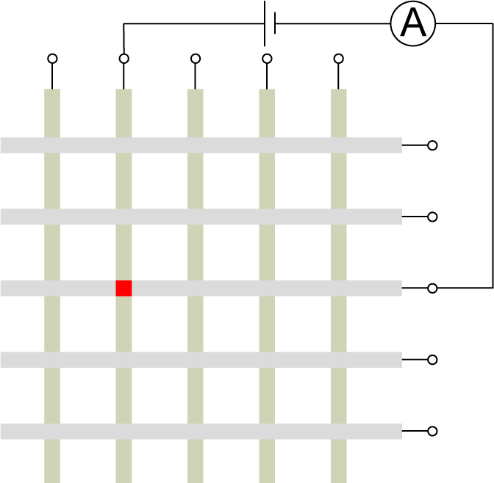


Fig. S19. Schematic illustration of measurement circuit about a 5×5 pixels photodetector array.

Table S1. Comparison of trap density between the previous reports and our work.

| Materials | Method | Trap density (cm^-3^) | Ref. |
| --- | --- | --- | --- |
| MAPbX_3_  MWs | Capillary-assisted method | 1.9 × 10^13^ | 7 |
| CsPbBr_3_  MPs | Inkjet printing and vapor phase growth | 2.1 × 10^12^ | 8 |
| MAPbBr_3_  MWs | Capillary-assisted method | 3.2 × 10^11^ | 9 |
| MAPbBr_3_  MWs | PDMS template-assisted method | 1.61 × 10^11^ | 10 |
| (R-MBA)_2_PbI_4_/  (S-MBA)_2_PbI_4_  NWs | Template-assisted and capillary-assisted method | 9.1 × 10^12^ | 11 |
| CsPbBr_3_  NWs | Capillary-assisted method | 1.06 × 10^12^ | 12 |
| MAPbBr_3_  MPs | Space confinement and antisolvent-assisted crystallization | 1.05 × 10^12^ | This work |

MW: microwire, MP: microplate, NW: nanowire

Table S2. Comparison of threshold and Q factor between the previous reports and our work.

| Materials | Method | Threshold (μJ cm^-2^) | Q factor | Array configuration | Ref. |
| --- | --- | --- | --- | --- | --- |
| MAPbBr_3_  MPs | Liquid knife | 3.5 | 1090 | Y | 13 |
| MAPbBr_3_  MPs | One-step solution | 9.1 | 2605 | N | 14 |
| MAPbBr_3_  microrods | Liquid-phase self-assembly | 62 | 657 | N | 15 |
| MAPbBr_3_  NWs | PDMS rectangular groove-templates | 12.3 | 500 | Y | 16 |
| MAPbBr_3_  NWs | Solution-processed | 9.8 | 1260 | N | 17 |
| MAPbBr_3_  thin film | Spin-coating | 16.2 | 1570 | Y | 18 |
| MAPbBr_3_  thin film | Spin-coating | 2.75 | 1025 | Y | 19 |
| CsPbBr*_x_*I_3-_*_x_*  NWs | Vapor phase epitaxial growth | 28 | 1390 | N | 20 |
| CsPbBr_3_  MPs | Vapor phase growth | 4.0 | 6806 | Y | 8 |
| CsPbBr_3_  MWs | Template-confined antisolvent crystallization | 2.2 | 1555 | Y | 2 |
| CsPbBr_3_  rings | Novel self-healing lithographic patterning | 3.8 | 2200 | Y | 21 |
| MAPbBr_3_  MPs | Space confinement and antisolvent-assisted crystallization | 4.14 | 2915 | Y | This work |

MP: microplate, NW: nanowire, MW: microwire

**REFERENCES**

1. Chen, J. *et al.* Single-crystal thin films of cesium lead bromide perovskite epitaxially grown on metal oxide perovskite (SrTiO_3_). *Journal of the American Chemical Society* **139**, 13525-13532 (2017).
2. Yang, Z. *et al.* Controllable growth of aligned monocrystalline CsPbBr_3_ microwire arrays for piezoelectric-induced dynamic modulation of single-mode lasing. *Advanced Materials* **31**, 1800596 (2019).
3. Zhu, Y. *et al.* Inhomogeneous trap-state-mediated ultrafast photocarrier dynamics in CsPbBr_3_ microplates. *ACS Applied Materials & Interfaces* **13**, 6820-6829 (2021).
4. Zhu, Y. *et al.* Spatiotemporal sectioning of two-photon fluorescence ellipsoid with a CsPbBr_3_ nanosheet. *Nano Research* **14**, 4288-4293 (2021).
5. Shi, L. *et al.* Status and outlook of metal-inorganic semiconductor-metal photodetectors. *Laser & Photonics Reviews* **15**, 2000401 (2021).
6. Liu, C. H. *et al.* Graphene photodetectors with ultra-broadband and high responsivity at room temperature. *Nature Nanotechnology* **9**, 273-278 (2014).
7. Gao, H. *et al.* Bandgap engineering of single-crystalline perovskite arrays for high-performance photodetectors. *Advanced Functional Materials* **28**, 1804349 (2018).
8. Gu, Z. *et al.* Controllable growth of high-quality inorganic perovskite microplate arrays for functional optoelectronics. *Advanced Materials* **32**, 1908006 (2020).
9. Li, S. X. *et al.* Curved photodetectors based on perovskite microwire arrays via in situ conformal nanoimprinting. *Advanced Functional Materials* **32**, 2202277 (2022).
10. Li, S. X. *et al.* Perovskite single-crystal microwire-array photodetectors with performance stability beyond 1 year. *Advanced Materials* **32**, 2001998 (2020).
11. Liu, Z. *et al.* Chiral hybrid perovskite single-crystal nanowire arrays for high-performance circularly polarized light detection. *Advanced Science* **8**, 2102065 (2021).
12. Pan, S. *et al.* Rapid capillary-assisted solution printing of perovskite nanowire arrays enables scalable production of photodetectors. *Angewandte Chemie International Edition* **59**, 14942-14949 (2020).
13. Feng, J. *et al.* "Liquid Knife" to fabricate patterning single-crystalline perovskite microplates toward high-performance laser arrays. *Advanced Materials* **28**, 3732-3741 (2016).
14. Wang, K. *et al.* Single-crystalline perovskite microlasers for high-Contrast and sub-Diffraction imaging. *Advanced Functional Materials* **29**, 1904868 (2019).
15. Zhang, W. *et al.* Controlling the cavity structures of two-photon-pumped perovskite microlasers. *Advanced Materials* **28**, 4040-4046 (2016).
16. Liu, P. *et al.* Organic-inorganic hybrid perovskite nanowire laser arrays. *ACS Nano* **11**, 5766-5773 (2017).
17. He, X. *et al.* Multi-color perovskite nanowire lasers through kinetically controlled solution growth followed by gas-phase halide exchange. *Journal of Materials Chemistry C* **5**, 12707-12713 (2017).
18. Wang, K. *et al.* Wettability-guided screen printing of perovskite microlaser arrays for current-driven displays. *Advanced Materials* **32**, 2001999 (2020).
19. Duan, Z. *et al.* Chip-scale fabrication of uniform lead halide perovskites microlaser array and photodetector array. *Laser & Photonics Reviews* **12**, 1700234 (2018).
20. Huang, L. *et al.* Composition-graded cesium lead halide perovskite nanowires with tunable dual-color lasing performance. *Advanced Materials* **30**, 1800596 (2018).
21. Xing, D. *et al.* Self-healing lithographic patterning of perovskite nanocrystals for large-area single-mode laser array. *Advanced Functional Materials* **31**, 2006283 (2021).
